# Gestational weight gain among American Samoan women and its impact on delivery and infant outcomes

**DOI:** 10.1186/s12884-015-0451-1

**Published:** 2015-02-03

**Authors:** Nicola L Hawley, William Johnson, Chantelle N Hart, Elizabeth W Triche, John Ah Ching, Bethel Muasau-Howard, Stephen T McGarvey

**Affiliations:** Department of Chronic Disease Epidemiology, School of Public Health, Yale University, P.O. Box 208034, New Haven, CT 06520-8034 USA; MRC Human Nutrition Research Unit, Cambridge, UK; Center for Obesity Research and Education & the Department of Public Health, Temple University, Philadelphia, USA; Department of Epidemiology, School of Public Health, Brown University, Providence, USA; LBJ Tropical Medical Center, Pago Pago, American Samoa USA; International Health Institute, School of Public Health, Brown University, Providence, USA

**Keywords:** American Samoa, Birth size, Cesarean delivery, Gestational weight gain, Obesity

## Abstract

**Background:**

As obesity has increased worldwide, so have levels of obesity during pregnancy and excess gestational weight gain (GWG). The aim of this paper was to describe GWG among American Samoan women and examine the association between GWG and four adverse pregnancy and infant outcomes: cesarean delivery, small- and large-for-gestational age (SGA/LGA), and infant overweight/obesity.

**Methods:**

Data were extracted from prenatal care records of 632 Samoan women. Mixed-effects growth models were used to produce individual weight-for-gestational week curves from which second and third trimester weight gain was estimated. Binary logistic regression was used to examine associations between GWG and the outcomes of interest.

**Results:**

Most women were overweight/obese in early pregnancy (86%) and 78% exceeded the Institute of Medicine GWG guidelines. Greater GWG in the second trimester and early pregnancy weight were independently associated with increased odds of a c-section (OR 1.40 [95% CI: 1.08, 1.83]) and OR 1.51 [95% CI: 1.17, 1.95], respectively). Risk of delivering a LGA infant increased with greater third trimester weight gain and higher early pregnancy weight, while second trimester weight gain was negatively associated with SGA. Risk of infant overweight/obesity at 12 months increased with early pregnancy weight (OR: 1.23 [95% CI: 1.01, 1.51]) and infant birthweight.

**Conclusions:**

The high levels of pregnancy obesity and excessive GWG in American Samoa suggest that it is important for physicians to encourage women into prenatal care early and begin education about appropriate GWG and the potential risks of excess weight gain for both the mother and baby.

## Background

As obesity has increased worldwide, so have levels of obesity during pregnancy and excess gestational weight gain (GWG) [1-3]. Excess GWG is associated with numerous adverse outcomes, including gestational diabetes mellitus (GDM), preeclampsia, early pregnancy loss, and post-partum weight retention [4,5]. Pre-pregnancy obesity is independently and additively associated with the same outcomes [6]. Additionally, there are long-term implications of high pre-pregnancy body mass index (BMI) and excess GWG for offspring overweight and associated metabolic disorders [7,8].

Contemporary Pacific Islanders have high BMI levels as a result of modernization and nutrition transition [9]. In American Samoa obesity is strikingly prevalent. Among women of childbearing age (18–44 years), 71% are obese according to Polynesian-specific BMI cutoffs (BMI ≥32 kg/m^2^), with a further 19% overweight (BMI 26–31.9 kg/m^2^) [10,11]. However, little is known about GWG among Samoan women and its impact on pregnancy and infant outcomes [12].

This paper describes GWG among American Samoan women and its association with maternal socio-demographic characteristics. Among a sub-sample of women, we document pregnancy obesity and the proportion of women exceeding the Institute of Medicine (IOM) GWG guidelines [3]. Additionally, this paper will examine the association between GWG and four adverse outcomes (c-section, small- and large-for gestational age (SGA/LGA), and offspring overweight/obesity at 12 months of age), observing differential effects of second and third trimester weight gain on these outcomes.

## Methods

### Setting

American Samoa lies ~2,400 miles southwest of Hawaii and ~1,800 miles northeast of New Zealand. Of the population of 54,517, 92% are native Samoans who are recognized as US nationals [13]. With a gross domestic product of $8,000 (2012) American Samoa is classified as an upper-middle income country [14] although, relative to US standards, more than half of families have incomes below the poverty line [15]. Prenatal care is delivered predominantly at the Lyndon B Johnson Tropical Medical Center (LBJTMC), the only full service hospital, where 97% of the ~1300 births each year occur.

### Data

**Figure 1:**
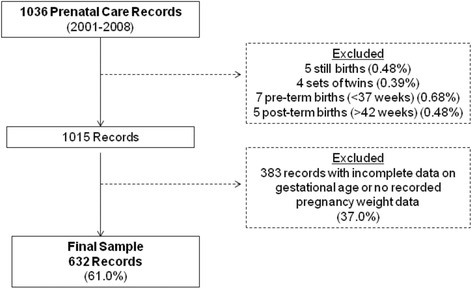
**Analysis sample selection.**

Data were extracted during a review of LBJTMC prenatal care records from 2001–2008. Of the 1036 records available for review, 632 (61%) were included in these analyses based on women being Samoan, having singleton, term pregnancies (37–42 weeks gestation), and weight measurements during pregnancy (Figure 1). There were no socio-demographic differences (age, marital status, education) between those included and excluded from the analyses based on these criteria.

Records were reviewed to determine weight and week of gestation at each prenatal visit. While weight is a standard prenatal care measure in American Samoa, height is not, therefore height (and consequently BMI) was only available for 31.6% of the sample (n = 200). There were no socio-demographic differences between women with height measurements and the larger sample; there were also no differences in early pregnancy weight (13 weeks), total GWG or prevalence of adverse study outcomes. Women for whom BMI was calculated were classified using Polynesian-specific cut-offs for overweight (BMI ≥26 kg/m^2^) and obesity (BMI ≥32 kg/m^2^). These Polynesian-specific cutoffs, derived from gold standard body composition assessments, were designed to identify the cut-points of percent body fat in this population which correspond to the WHO standards of BMI 25 and 30 kg/m^2^ [10].

As pre-pregnancy weight was not recorded in the prenatal records, BMI was calculated at 13 weeks and is therefore referred to as “early pregnancy BMI”. Using this early pregnancy BMI, women were classified as gaining under, within, or over the IOM guidelines. The IOM recommend normal weight women gain 11.4-15.9 kg, overweight women 6.8-11.4 kg, and obese women 5.0-9.1 kg [3].

Mode of delivery, infant birthweight, number of prenatal care visits and socio-demographic characteristics of the participants (Table 1) were also recorded. Infants were classified at birth as SGA (<10th percentile), appropriate-for-gestational age (AGA), or LGA (>90th percentile) based upon the CDC 2000 US growth references (there are no Polynesian-specific references for infant size) [16].

**Table 1:** **Maternal & infant characteristics and their association with gestational weight gain**

**Maternal characteristic**	**Subjects n (%)**	**Gestational weight gain 13–40 weeks, kg (mean ± SD)**
Maternal age (years)**	**632**	
<20 years	34 (5.4)	17.38 ± 7.15
20 – 29 years	350 (55.4)	15.34 ± 5.42
30 – 39 years	230 (36.4)	13.47 ± 4.96
>40 years	18 (2.8)	12.20 ± 4.12
**Education level**	**283**	
Less than High School	45 (15.9)	13.71 ± 4.53
Completed High School	186 (65.7)	14.46 ± 4.96
College/Higher Education	52 (18.4)	15.34 ± 5.90
Marital status*	**609**	
Single, Divorced, Widowed, Separated	112 (18.4)	15.66 ± 6.97
Married, Cohabiting, Partner	497 (81.6)	14.47 ± 5.08
Maternal occupation**	**614**	
Unemployed	361 (58.8)	14.26 ± 5.18
Professional/Managerial/Sales	73 (11.6)	16.87 ± 6.20
Service/elementary occupations	180 (29.3)	14.75 ± 5.55
Parity**	**632**	
Primiparous	123 (19.5)	17.50 ± 6.39
1- 4 children	335 (53.0)	14.49 ± 5.01
≥4 children	174 (27.5)	13.05 ± 4.72
**Early pregnancy weight (13 weeks)**	**632**	
<60 kgs	44 (7.0)	14.51 ± 4.33
60 – 80 kgs	241 (38.1)	14.98 ± 5.63
80 – 100 kgs	232 (36.7)	14.34 ± 5.14
100 – 120 kgs	92 (14.6)	15.08 ± 6.08
>120 kgs	23 (3.6)	13.69 ± 5.79
**Number of prenatal care visits**	**632**	
<4	129 (20.4)	14.12 (3.68)
4 - 7	255 (40.3)	14.68 (5.58)
8 – 12	207 (32.8)	14.66 (5.61)
>12	41 (6.5)	16.47 (7.76)
Delivery type**	**597**	
Vaginal	529 (88.6)	14.41 ± 4.97
Caesarian Section	68 (11.4)	16.77 ± 8.20
Birthweight for gestational age*	**632**	
Small (<10th percentile)	28 (4.4)	12.78 ± 5.13
Normal	506 (80.1)	14.58 ± 5.20
Large (>90th percentile)	98 (15.5)	15.75 ± 6.53
**Infants size at 12 months**	**517**	
Normal (Weight-for-length <85th percentile)	350 (67.7)	14.53 ± 5.34
Overweight (85th < 95th percentile)	79 (15.3)	14.55 ± 5.50
Obese (≥95th percentile)	88 (17.0)	15.48 ± 6.26
**Infant sex**	**517**	
Male	271 (52.4)	14.38 ± 5.03
Female	246 (47.6)	15.04 ± 6.06

Main effects significant: *p < 0.05; **p < 0.01.

Of the 632 mother-infant dyads whose prenatal care/birth data were included in this analysis, 517 infants were included in a prior analysis of infant growth patterns [17]. Therefore, we were able to examine the association of GWG with infant weight status at 12 months in these 517 infants (normal weight [<85th weight-for-length percentile], overweight/obese [≥85th percentile]) [16].

Data collection was approved by the institutional review boards at Brown University, the American Samoa Department of Health, and the LBJTMC Privacy Office.

### Modeling gestational weight gain

A mixed-effects growth model was applied to serial maternal weight data from 13 to 42 weeks of gestation to produce individual weight-for-gestational week curves using *xtmixed* in Stata 12.0 (StataCorp. 2011. College Station, TX). Data before 13 weeks gestation (n = 78 observations from 57 pregnancies) were too sparse to include. There were 4175 weight measurements included in the model (mean = 6.6 per woman) over an average period of 12.6 weeks. Change in weight was modeled as a restricted cubic regression spline of age, specifying five knots at equally spaced quantiles of the gestational week distribution. The intercept and all four gestational week terms were allowed to have random (as well as fixed) effects, an unstructured variance-covariance matrix was specified, and the model was estimated using maximum likelihood. After fitting the model once, observations with residuals >1.5 kg or <1.5 kg (n = 114 from 83 women) were removed on the assumption that they included large measurement error, and the model was fitted a second time. The final model provided an excellent fit for the data as assessed by the residual standard deviation (0.66 kg) and Bayesian information criterion (16133). The model was used to estimate weight at 13, 28, and 40 weeks for each individual, and these estimates used in secondary analyses.

### Statistical analysis

Independent samples t-tests, ANOVA, and Chi-squared (X^2^) tests were used to examine differences in GWG according to maternal socio-demographic characteristics and the study outcomes.

Stepwise binary logistic regression was used to examine the association between GWG and the outcomes of interest. Gestational weight gain, maternal age and parity, and estimated maternal weight at 13 weeks gestation were included in each of the regression models. The infant overweight/obesity model also included infant birth weight. To explore whether weight gained in the second trimester (13–28 weeks) had a differential effect on study outcomes compared to weight gained in the third trimester (28–40 weeks) we used conditional weight gain variables to deal with the tendency of second and third trimester weight gain to be highly correlated in the same individual (r = 0.524, p < 0.001 in our study sample). The conditional variables for each trimester represent standardized residuals from regressions of weight at one time point on weight at all previous time points (i.e. weight at 28 weeks on weight at 13 weeks). By design, these conditional variables are uncorrelated with each other (and were also uncorrelated with weight at age 13 weeks) and therefore can be included in a single multivariable model without causing variance inflation. Interactions between the GWG variables and early pregnancy weight were explored in each model. For ease of interpretation, beta values from the models are presented per standard deviation change in the predictor.

Sensitivity analyses were conducted in the sub-sample for whom height was available, replacing early pregnancy weight with early pregnancy BMI. Statistical analyses were undertaken using SPSS version 18.0 (SPSS Inc., Illinois, USA) and SAS version 9.3 (SAS Institute Inc., Cary, NC, USA).

## Results

### Sample characteristics

Mean GWG between 13 and 40 weeks was 14.67 ± 5.29 kgs (13–28 weeks: 7.85 ± 3.12; 28–40 weeks: 6.78 ± 2.94).

GWG was significantly lower in older, more parous women, with age and parity strongly related (r = 0.629, p < 0.001) (Table 1). Married women gained less weight between 13 and 40 weeks than single women, although this likely reflects associations between marital status, age, and parity.

Among the sub-sample for whom we were able to calculate early pregnancy BMI the majority were overweight/obese (86%) at 13 weeks (Table 2). Mean early pregnancy BMI was 31.6 kg/m^2^ (range: 17.33 to 58.04 kg/m^2^). The vast majority of women (78%) exceeded IOM guidelines for GWG. GWG did not significantly differ according to early pregnancy BMI (F = 0.67, p = 0.52), resulting in significantly more overweight and obese women exceeding the guidelines compared to normal weight (χ^2^ = 21.08, p < 0.001) (based on their lower GWG goals).

**Table 2:** **Adherence to IOM guidelines for gestational weight gain based upon early pregnancy BMI (sub-sample of women with recorded height only, n = 200)**

			**Adherence to IOM guidelines for gestational weight gain**		
**Early pregnancy BMI (13 weeks)**	**n**	**Mean gestational weight gain (kgs)**	**Under recommended gain n (%)**	**Within IOM guidelines n (%)**	**Above recommended gain n (%)**
Normal weight (<26 kg/m^2^)	26	16.33 ± 6.01	4 (14.3)	11 (39.3)	13 (46.4)
Overweight (26 - < 32 kg/m^2^)	68	15.13 ± 4.68	3 (4.4)	12 (17.6)	53 (77.9)
Obese (>32 kg/m^2^)	104	15.09 ± 5.34	3 (2.9)	11 (10.6)	90 (86.5)
**Total**	**200**	**15.28 ± 5.22**	**10 (5.0)**	**34 (17.0)**	**156 (78.0)**

Institute of Medicine guidelines for gestational weight gain: Normal weight 11.4-15.9 kg; Overweight 6.8-11.4 kg; Obese 5.0-9.1 kg. BMI cut-offs used here are Polynesian specific cut-offs which correspond to the WHO standards of BMI 25 and 30 kg/m^2^ for overweight and obesity respectively.

In fully adjusted logistic regression models, greater GWG in the second trimester and early pregnancy weight were independently associated with increased odds of a c-section (OR 1.40 [95% CI: 1.08, 1.83]) and OR 1.51 [95% CI: 1.17, 1.95], respectively; ORs per SD change in the predictor). There was a significant interaction between second trimester (13–28 week) GWG and early pregnancy weight, with a multiplicative effect on the outcome (data not shown). There was an interaction of a similar magnitude between third trimester GWG and early pregnancy weight, although there was no interaction between GWG in each of the trimesters (Table 3).

**Table 3:** **Risk of cesarean section based upon gestational weight gain**

		**OR** ^**†**^ **c-section vs. vaginal delivery (95% CI)**			
**Predictor**	**SD**	**0**	**1**	**2**	**3**
Gestational weight gain^‡^ (13–28 weeks) (z-score)	1.00	1.43 (1.10, 1.86)	1.43 (1.10, 1.86)	1.40 (1.08, 1.82)	1.40 (1.08, 1.83)
Gestational weight gain^‡^ (28–40 weeks) (z-score)	1.00	1.06 (0.80, 1.41)	1.12 (0.84, 1.51)	1.13 (0.84, 1.50)	1.13 (0.84, 1.52)
Maternal age (years)	5.57	-	1.18 (0.85, 1.64)	1.16 (0.84, 1.61)	1.16 (0.82, 1.14)
Parity	N/A	-	1.01 (0.86, 1.19)	0.97 (0.82, 1.14)	0.97 (0.67, 1.31)
Estimated weight at 13 weeks (kg)	17.71	-	-	1.51 (1.17, 1.94)	1.51 (1.17, 1.95)
Infant birthweight (g)	493.57				0.97 (0.74, 1.27)

^†^OR are presented per SD change in predictors (except parity); c-section: cesarean section; ^‡^conditional weight gain variables.

Risk of delivering a LGA infant increased with greater third trimester weight gain and higher early pregnancy weight. There were no significant interactions between early pregnancy weight and GWG in either trimester. Only second trimester GWG independently predicted SGA risk with 40% less risk per SD increase in GWG. Again, there were no significant interactions between GWG and early pregnancy weight, although there was a significant interaction between GWG in the second and third trimester; poor weight gain in both trimesters multiplied SGA risk (Table 4 and 5).

**Table 4:** **Risk of delivering a LGA baby based upon gestational weight gain**

		**OR** ^**†**^ **LGA vs. AGA (95% CI)**		
**Predictor**	**SD**	**0**	**1**	**2**
Gestational weight gain^‡^ (13–40 weeks) (z-score)	1.00	1.11 (0.87, 1.42)	1.12 (0.88, 1.43)	1.09 (0.85, 1.40)
Gestational weight gain^‡^ (28–40 weeks) (z-score)	1.00	1.15 (0.90, 1.47)	1.28 (0.98, 1.65)	1.29 (1.00, 1.67)
Maternal age (years)	5.54	-	1.25 (0.95, 1.65)	1.23 (0.93, 1.63)
Parity	N/A	-	1.07 (0.94, 1.23)	1.03 (0.90, 1.18)
Estimated weight at 13 weeks (kg)	17.73	-	-	1.42 (1.14, 1.77)

^†^OR are presented per SD change in predictors (except parity); LGA: large-for-gestational age, AGA: appropriate-for-gestational age.

^‡^Conditional weight gain variables.

**Table 5:** **Risk of delivering a SGA baby based upon gestational weight gain**

		**OR** ^**†**^ **SGA vs. AGA (95% CI)**		
**Predictor**	**SD**	**0**	**1**	**2**
Gestational weight gain^‡^ (13–28 weeks) (z-score)	1.00	0.59 (0.37, 0.96)	0.58 (0.36, 0.96)	0.60 (0.36, 0.99)
Gestational weight gain^‡^ (28–40 weeks) (z-score)	1.00	1.05 (0.66, 1.67)	0.93 (0.57, 1.51)	0.91 (0.55, 1.51)
Maternal age (years)	5.54	-	0.77 (0.46, 1.28)	0.81 (0.49, 1.36)
Parity	N/A	-	0.88 (0.67, 1.17)	0.92 (0.69, 1.22)
Estimated weight at 13 weeks (kg)	17.73	-	-	0.67 (0.43, 1.06)

^†^OR are presented per SD change in predictors (except parity); SGA: small-for-gestational age, AGA: appropriate-for-gestational age.

^‡^Conditional weight gain variables.

GWG was not associated with risk of infant overweight/obesity at 12 months. Risk of infant overweight/obesity increased with early pregnancy weight (OR: 1.23 [95% CI: 1.01, 1.51) and infant birthweight (88% greater risk per 475.23 g (1SD) increase in birthweight). There were no significant interactions between GWG and early pregnancy weight (Table 6).

**Table 6:** **Risk of infant overweight/obesity (weight-for-length ≥85th percentile) at 12 months based upon gestational weight gain**

		**OR** ^**†**^ **infant being overweight/obese vs. normal weight at 12 months (95% CI)**			
**Predictor**	**SD**	**0**	**1**	**2**	**3**
Gestational weight gain^‡^ (13–28 weeks) (z-score)	1.00	0.98 (0.79, 1.22)	0.98 (0.79, 1.22)	0.96 (0.77, 1.21)	0.91 (0.72, 1.16)
Gestational weight gain^‡^ (28–40 weeks) (z-score)	1.00	1.13 (0.91, 1.41)	1.09 (0.87, 1.37)	1.09 (0.87, 1.38)	1.00 (0.79, 1.28)
Maternal age (years)	5.63	-	0.97 (0.76, 1.23)	0.94 (0.74, 1.19)	0.88 (0.68, 1.13)
Parity	N/A	-	0.96 (0.85, 1.08)	0.93 (0.82, 1.05)	0.90 (0.79, 1.02)
Estimated weight at 13 weeks (kg)	17.86	-	-	1.34 (1.10, 1.62)	1.23 (1.01, 1.51)
Infant birthweight (g)	475.23	-	-	-	1.88 (1.51, 2.33)

^†^OR are presented per SD change in predictors (except parity); ^‡^conditional weight gain variables.

In sensitivity analyses, logistic regressions were repeated in the sub-sample for whom height was available (n = 200) and early pregnancy weight replaced with early pregnancy BMI. Some associations were attenuated by the reduced sample size but the ORs retained their direction and magnitude (data not shown). We also examined whether adherence to IOM guidelines was associated with study outcomes and detected no difference in the risk for any of the outcomes according to adherence to the IOM guidelines.

## Discussion

This paper is the first to describe GWG among women in American Samoa, an important population to examine given the extremely high population prevalence of obesity. Furthermore, we used an advanced technique to model non-consistently collected pregnancy weight data, allowing us to examine the differential associations of second and third trimester weight gain with risk of c-section, size-for-gestational age of the infant, and offspring overweight/obesity at 12 months of age.

There were high levels of early pregnancy overweight and obesity (86% combined [Polynesian BMI cut-offs]; 52% obese) among the study sample and the vast majority of women (78%) exceeded the current IOM guidelines for GWG. It should be noted, however, that because these data were collected prior to the publication of the 2009 guidelines, obese women in particular may have been counselled to gain a different amount of weight than is currently recommended.

The absolute magnitude of weight gain reported here is similar to that reported across other populations [18-20]; however, we were restricted to describing weight gained between 13 and 40 weeks rather than considering pre-pregnancy weight. As a result, 78% of women exceeding IOM guidelines may be a conservative estimate. Evidence from a multi-ethnic US cohort suggested that first trimester weight gain is minimal (1–4 lbs) [21] but this may not be the case in a population with such propensity for obesity and excessive GWG. Further research is needed to determine the magnitude of first trimester weight gain and its association with maternal and infant outcomes.

GWG was significantly less in older, married, more parous women, a finding consistent with the existing literature [19], although in this sample this was offset by the fact that early pregnancy weight was much greater in multiparous than primiparous women (mean early pregnancy weight: primiparous = 78.2 kgs, parity ≥4 = 90.7 kgs). Being employed in a higher level occupation was associated with greater GWG, likely reflecting higher income and potentially easier access to high-fat, non-traditional foods.

In this study, second trimester (13–28 week) GWG was positively associated with risk of c-section and negatively associated with SGA. In an earlier study of European, non-obese women, second trimester weight gain had a greater impact on birthweight than either first or third trimester gain [22]; each kilogram of weight gain during the second trimester was associated with a 32.8 g increase in birthweight, compared with 18.0 g/kg in the first trimester and 17.0 g/kg in the third. Many of the maternal physiological changes in the second trimester (uterus expansion, increasing blood supply, creation of fat stores) [23,24] support later fetal growth, therefore lower second trimester gain may be synonymous with poorer physiological preparation of the mother to support the pregnancy and consequently SGA in the infant. Similarly, these same physiological insufficiencies in the mother may lead to complications that necessitate c-section, although the specific clinical indicators for c-sections in this sample were not documented.

Third trimester GWG (28–40 weeks), and not second trimester gain, was positively associated with risk of a LGA baby in this study. This is in line with third trimester nutritional intake (reflected in gestational weight gain) being largely directed toward the fetus as it undergoes the most rapid period of *in utero* weight and fat gain [25]. Equally, the third trimester gain may directly represent the additional growth of the fetus, which would need to be confirmed with ultrasound fetal size monitoring. It is important to recognize, however, that these documented relationships between GWG and infant size may not be causal. Both predictor and outcome may be linked to other pregnancy conditions (GDM, pre-eclampsia) which were not documented here.

Several prior studies have examined the association between excess GWG and offspring obesity risk, and a recent systematic review concluded that excess GWG does increase offspring obesity risk [26]. Our analyses do not support the association between GWG and offspring overweight/obesity risk. Instead we found that maternal early pregnancy weight (consistent with prior literature) and infant birthweight were the significant predictors of overweight/obesity at 12 months of age. This is, however, one of the first studies to explore offspring obesity very early in life, other studies have focused on children two years or older [26] and it may be that this association does not appear until later in life. Additionally, because of high maternal BMI it may be more difficult to detect the effects of GWG variation on offspring obesity.

This study had several limitations. Firstly, the analysis sample was limited to those for whom gestational dating and pregnancy weight data were available. Secondly, as women tended to enroll in prenatal care later in pregnancy our mixed-effects model of GWG was limited by the lack of data before 13 weeks, resulting in the need to constrain weight gain between 13 and 18 weeks to be linear. Biologically, weight gain is unlikely to be linear during any period of gestation, however the model residuals showed a good model fit, with residuals for 13–18 weeks no greater than for any other period of gestation. Thirdly, our two-step approach, using the model estimates in secondary analyses, potentially increased type I error in the secondary models. Although it was possible to fit our growth model and relate the parameters to some distal outcome in a one-step approach this was not done because the parameters of the restricted cubic spline are not easily interpretable. Lastly, height was not commonly recorded in the prenatal records, so we were only able to calculate BMI in 200 of the 632 women. Sensitivity analyses, however, indicated that the study outcomes would have been the same but the influence of BMI vs. weight alone should be examined in future studies. In the future, recording height and BMI in prenatal care records in American Samoa would be beneficial to allow clinicians to give appropriate guidance for weight gain during pregnancy.

## Conclusions

The high levels of pregnancy obesity and excessive GWG reported here suggest that it is important for physicians in American Samoa and those treating Samoan populations across the Asia-Pacific to educate women about appropriate gestational weight gain. Attention should also be paid to pre-pregnancy BMI and advice given to women about appropriate pre-conception weight status.

## Abbreviations

BMI: Body Mass Index
GDM: Gestational Diabetes Mellitus
GWG: Gestational Weight Gain
IOM: Institute of Medicine
LGA: Large for gestational age
SGA: Small for gestational age

## References

[1] Callaway LK, Prins JB, Chang AM, McIntyre HD (2006). The prevalence and impact of overweight and obesity in an Australian obstetric population. Med J Aust.

[2] Fisher SC, Kim SY, Sharma AJ, Rochat R, Morrow B (2013). Is obesity still increasing among pregnant women? Prepregnancy obesity trends in 20 states, 2003–2009. Prev Med.

[3] Rasmussen KM, Yaktine AL, Institute of Medicine (US) and National Research Council (US) Committee to Reexamine IOM Pregnancy Weight Guidelines (2009). Weight gain during pregnancy: reexamining the guidelines.

[4] Siega-Riz AM, Viswanathan M, Moos MK, Deierlein A, Mumford S, Knaack J (2009). A systematic review of outcomes of maternal weight gain according to the Institute of Medicine recommendations: birthweight, fetal growth, and postpartum weight retention. Am J Obstet Gynecol.

[5] Mamun AA, Callaway LK, O’Callaghan MJ, Williams GM, Najman JM, Alati R (2011). Associations of maternal pre-pregnancy obesity and excess pregnancy weight gains with adverse pregnancy outcomes and length of hospital stay. BMC Pregnancy Childbirth.

[6] Catalano PM, Ehrenberg HM (2006). The short- and long term implications of maternal obesity on the mother and her offspring. Brit J Obstet Gynaec.

[7] Wrotniak BH, Shults J, Butts S, Stettler N (2008). Gestational weight gain and risk of overweight in the offspring at age 7 y in a multicenter, multiethnic cohort study. Am J Clin Nutr.

[8] Oken E, Rifas-Shirman SL, Field AE, Frazier AL, Gillman MW (2008). Maternal gestational weight gain and offspring weight in adolescence. Obstet Gynecol.

[9] McGarvey ST (1991). Obesity in Samoans and a perspective on its etiology in Polynesians. Am J Clin Nutr.

[10] Swinburn BA, Ley SJ, Carmicheal HE, Plank LD (1999). Body size and composition in Polynesians. Int J Obes.

[11] Keighley ED, McGarvey ST, Quested C, McCuddin C, Viali S, Maga UA, Ohtsuka R, Ulijasek SJ (2007). Nutrition and health in modernizing Samoas: temporal trends and adaptive perspectives. Health change in the Asia-Pacific region: biocultural and epidemiological approaches.

[12] Hawley NL, Brown C, Nu’usolia O, Ah Ching J, Muasau-Howard B, McGarvey ST. Barriers to adequate prenatal care utilization in American Samoa. Matern Child Health J. in press. 10.1007/s10995-013-1368-9 PMC3959630 24045912

[13] US Central Intelligence Agency. The World Factbook. [https://www.cia.gov/library/publications/the-world-factbook/geos/aq.html]

[14] The World Bank. [http://data.worldbank.org/income-level/UMC 2014]

[15] American Samoa Department of Commerce. American Samoa at-a-glance. [www.spc.int/prism/country/as/stats/At_A_Glance_2006.pdf 2006]

[16] Kuczmarski RJ, Ogden CL, Grummer-Strawn LM, Flegel KM, Guo SS, Wei R (2000). CDC Growth Charts: United States. Adv Data.

[17] Hawley NL, Johnson W, Nu’usolia O, McGarvey ST (2014). The contribution of feeding mode to obesogenic growth trajectories in American Samoan infants. Pediatr Obes.

[18] Weisman CS, Hillemeier MM, Downs DS, Chuang CH, Dyer AM (2010). Preconception predictors of weight gain during pregnancy: prospective findings from the central Pennsylvania women’s health study. Womens Health Issues.

[19] Chasan-Taber L, Schmidt MD, Pekow P, Sternfeld B, Solomon CG, Markenson G (2008). Predictors of excessive and inadequate gestational weight gain in Hispanic women. Obesity (Silver Spring).

[20] Kac G, Benicio MHDA, Valásquez-Meléndez G, Valente JG, Struchiner CJ (2004). Gestational weight gain and prepregnancy weight influence postpartum weight retention in a cohort of Brazilian women. J Nutr.

[21] Deierlein AL, Siega-Riz AM, Herring A (2008). Dietary energy density but not glycemic load is associated with gestational weight gain. AmJ Clin Nutr.

[22] Abrams B, Selvin S (1995). Maternal weight gain pattern and birth weight. Obstet Gynecol.

[23] Tan EK, Tan EL (2013). Alterations in physiology and anatomy during pregnancy. Best Pract Res Clin Obstet Gynaecol.

[24] Chandra S, Tripathi AK, Mishra S, Amzarul M, Vaish AK (2012). Physiological changes in haematological parameters during pregnancy. Indian J Hematol Blood Transfus.

[25] Mayer C, Joseph KS (2013). Fetal growth: a review of terms, concepts and issues relevant to obstetrics. Ultrasound Obstet Gynecol.

[26] Mamun AA, Mannan M, Doi SAR (2014). Gestational weight gain in relation to offspring obesity over the life course: a systematic review and bias-adjusted meta-analysis. Obes Rev.

